# Aberrant Expression of Bacterial Pattern Recognition Receptor NOD2 of Basophils and Microbicidal Peptides in Atopic Dermatitis

**DOI:** 10.3390/molecules21040471

**Published:** 2016-04-11

**Authors:** Chun-Kwok Wong, Ida Miu-Ting Chu, Kam-Lun Hon, Miranda Sin-Man Tsang, Christopher Wai-Kei Lam

**Affiliations:** 1 Department of Chemical Pathology, Prince of Wales Hospital, The Chinese University of Hong Kong, Shatin, NT, Hong Kong, China; idachu1219@gmail.com; 2 Institute of Chinese Medicine and State Key Laboratory of Phytochemistry and Plant Resources in West China, The Chinese University of Hong Kong, Hong Kong, China; ehon@cuhk.edu.hk (K.-L.H.); tsangsinman0128@gmail.com (M.S.-M.T.); 3 Department of Pediatrics, Prince of Wales Hospital, The Chinese University of Hong Kong, Shatin, NT, Hong Kong, China; 4 State Key Laboratory of Quality Research in Chinese Medicine, Macau University of Science and Technology, Macau, China; wklam@must.edu.mo

**Keywords:** antimicrobial peptides, atopic dermatitis, basophils, chemokines, pattern recognition receptors

## Abstract

Atopic dermatitis (AD) is a chronically relapsing inflammatory skin disease, associated with basophil infiltration into skin lesions and *Staphylococcus aureus* (*S.* *aureus*)-induced inflammation. Pattern recognition receptors (PRRs), including microbicidal peptide human neutrophil α-defensins (HNP) and dermcidin, can exert immunomodulating activity in innate immunity and skin inflammation. We investigated the plasma concentration of HNP and dermcidin, the expression of bacterial toll-like receptor (TLR) and nucleotide-binding oligomerization domain (NOD)-like receptors of basophils and plasma concentration and *ex vivo* induction of AD-related inflammatory cytokines and chemokines using ELISA and flow cytometry, in AD patients and control subjects. Plasma concentrations of HNP, dermcidin and AD-related Th2 chemokines CCL17, CCL22 and CCL27 were significantly elevated in AD patients compared with controls (all *p* < 0.05). Plasma concentrations of CCL27 and CCL22 were found to correlate positively with SCORing atopic dermatitis (SCORAD), objective SCORAD, % area affected, lichenification and disease intensity, and CCL27 also correlated positively with pruritus in AD patients (all *p* < 0.05). Protein expressions of NOD2 but not TLR2 of basophils were significantly down-regulated in AD patients compared with controls (*p* = 0.001). Correspondingly, there were lower *ex vivo* % inductions of allergic inflammatory tumor necrosis factor-α, IL-6 and CXCL8 from peripheral blood mononuclear cells upon NOD2 ligand *S. aureus* derived muramyl dipeptide stimulation in AD patients comparing with controls. The aberrant activation of bacterial PRRs of basophils and anti-bacterial innate immune response should be related with the allergic inflammation of AD.

## 1. Introduction

Atopic dermatitis (AD) is an important chronic relapsing inflammatory skin disease, especially in children, characterized by skin lesions with “lichenification”, pruritic excoriations and a susceptibility to cutaneous bacterial infections, that exhibits adverse impact on quality of life [1]. Basophils are rare circulating granulocytes (<1%) that contribute to the development of T helper (Th)2 immunity by elevated release of Th2 cytokines interleukin (IL)-4, IL-13, IL-17E/IL-25 and thymic stromal lymphopoietin (TSLP) in allergic inflammation [2]. Basophils therefore serve as initiators and accessory cells for the Th2 cell polarization in response to protease allergens and recruit other effector cells such as eosinophils or neutrophils [3,4]. This type of chronic allergic inflammation requires basophils as initiators, through the interaction of antigens, IgE and FcRI [3,4]. Basophils are rarely found in normal tissues. However, they have been detected in skin lesions of AD and urticaria but not in psoriasis vulgaris, mastocytosis or tumoral lesions [5]. Infiltrated basophils are essential for IgE-mediated chronic AD and for protection against secondary infections such as *N. brasiliensis* [6]. Basophils also produce leukotriene C4 and histamine which can cause the symptoms of acute and chronic allergic inflammation [4,5,6]. Patients with AD have a unique predisposition to colonization or infection by the Gram positive bacterial *Staphylococcus aureus* (*S. aureus*) [1,7]. *S. aureus* can exacerbate or contribute to persistent skin inflammation in AD by secreting toxins with superantigenic properties, resulting in marked activation of T cells and other immune cells [8]. Toll-like receptors (TLR), a group of innate immunity-related pattern recognition receptors (PRR) that mediated the activation of eosinophils and dermal fibroblasts cells in allergic inflammation, have been extensively studied [9,10,11]. Cell surface TLR2 on keratinocytes recognizing bacterial cell wall component peptidoglycan (PGN) of *S. aureus* contributes to the innate immune response via intracellular signaling transduction [12,13]. Cytosolic PRR nucleotide binding oligomerization domain (NOD) like receptors (NLR) sense the conserved bacterial structural components [14,15]. NOD2-dependent recognition of *S. aureus* derived muramyl dipeptide (MDP) can trigger the innate immunity-mediated inflammation [11,16].

Principal human neutrophil defensins (α-defensins)/human neutrophil peptide (HNP)1-3 belong to the family of cationic trisulfide-containing microbicidal peptides. HNP exerts chemotactic, immunomodulating and cytotoxic activity and participates in innate immunity and inflammation [17]. Expression of human β-defensin (HBD2) was found to be reduced in AD lesions as compared with normal controls or psoriasis [18]. Up-regulation of HNP1-3 in T cells from patients with Stevens-Johnson syndrome/toxic epidermal necrolysis was demonstrated to relate with the etiopathology of these life-threatening diseases induced by medications [19]. Another antimicrobial peptide dermcidin is an anionic peptide and expressed in exocrine sweat glands and transported via sweat to the epidermal surface, with potent antimicrobial activity against *S. aureus*, *Escherichia coli* and candida [20,21,22]. Unlike human cathelicidins and β-defensins which are inducible peptides in response to injury and inflammation, dermcidin is involved in the constitutive innate defence of human skin and not inducible by skin injury or inflammation [21].

Under inflammatory conditions in AD skin, decreased expression of antimicrobial peptide genes and dermcidin in sweat of AD patients with local up-regulation of Th2 cytokines and the lack of elevated amounts of TNF-α and interferon (IFN)-γ have been reported [20,23,24]. However, the circulating levels of HNP1-3 and dermcidin in AD patients have not yet been well elucidated. Our recent *in vitro* and animal study demonstrated that NOD2/TLR2-mediated exacerbation of AD can be through the activation of basophils interacting with dermal fibroblasts and therefore sheds light on a novel mechanistic pathway by which *S. aureus* contributes to the pathophysiology of AD [11]. These result prompted us to further evaluate the expression of TLR2 and NOD2 of circulating basophils in AD patients. To investigate the bacterial infection-mediated innate immunity in allergic inflammation, the expression and cytokine inducing function of TLR2 and NOD2 of basophils, circulating antimicrobial peptide HNP and dermcidin and the AD-related chemokines CCL17, CCL22 and CCL27 were evaluated in the present cross-sectional clinical study.

## 2. Results and Discussion

### 2.1. AD Patients and Control Subjects

The clinical characteristics of the study population are summarized in Table 1. Twenty-seven AD patients and 15 control subjects were enrolled in this study, sharing with similar age range. The severity and symptoms of AD were assessed and presented using different clinical parameters such as SCORing atopic dermatitis (SCORAD) index, objective SCORAD, pruritus, sleep loss, skin hydration (SH), transepidermal water loss (TWEL), % skin area affected, lichenification and Children’s dermatology life quality index (CDLQI) (Table 1). All AD patients were found to have specific IgE against allergen house dust mites *Dermatophagoides pteronyssinus* (*D. pteronyssinus*) and most of them also have specific IgE against at least one of the following allergens including mixed cockroaches, cat dander, dog dander, mixed molds and *Staphylococcal* enterotoxin A, B and C. None of the control subjects presented with specific IgE against the above allergens. Functional basophil activation test (BAT) results showed that none of the control subjects exhibited positive BAT, but positive BAT upon the challenge by house dust mite Der p1, cockroach Bla g 2 and cat dander Fel d 1 protein was found in 96%, 78% and 70%, respectively, of total AD patients. Among all 27 recruited AD patients, 24 (88.9%) and 27 (100%) received topical corticosteroid and emollient treatment, respectively. Results in Table 1 therefore mainly confirm the disease severity and symptoms, as well as the IgE-mediated allergen sensitization in AD patients. Using cellular functional assay of IgE, result of basophil activation allergenicity test was found to be in concordance with the results of serum assay of specific IgE.

### 2.2. Plasma Concentrations of AD-Related Chemokines and Bacterial Infection-Related Dermcidin and HNP 

**Figure 1:**
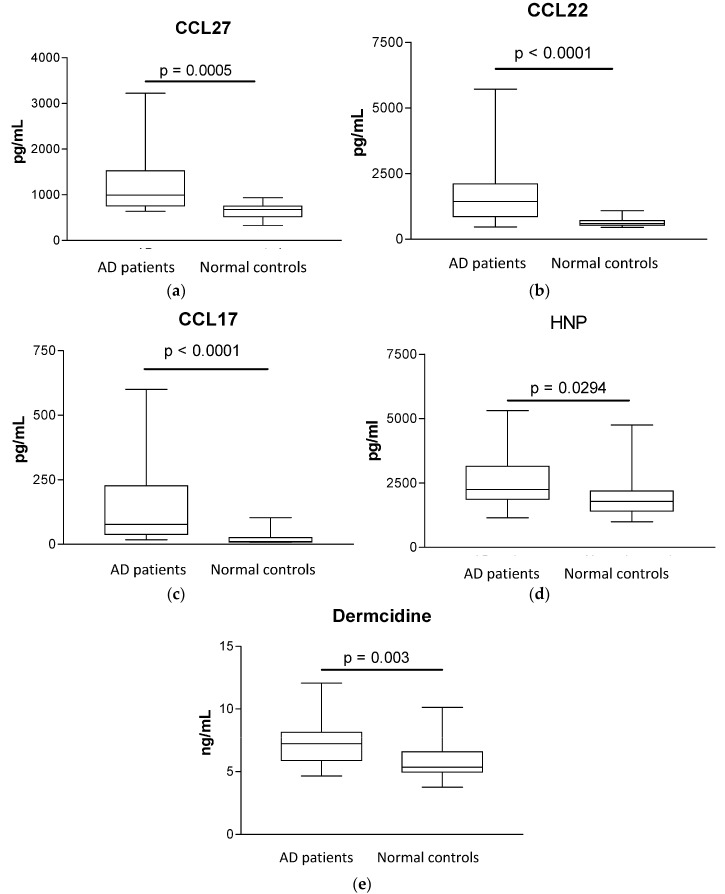
Plasma concentrations of CCL27, CCL22, CCL17, HNP and dermcidin in AD patients and normal controls. Box and whiskers plots showing comparisons of plasma concentrations of (**a**) CCL27; (**b**) CCL22; (**c**) CCL17; (**d**) HNP and (**e**) dermcidin between AD patients and control subjects were determined by Mann-Whitney rank sum test.

**Figure 2:**
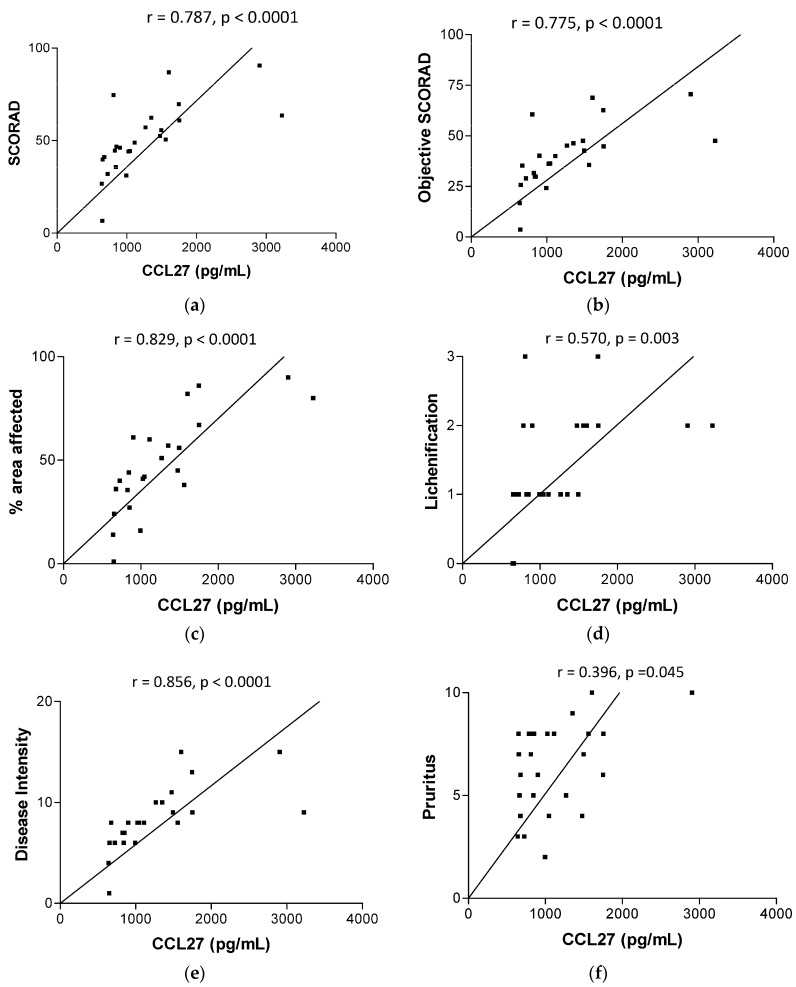

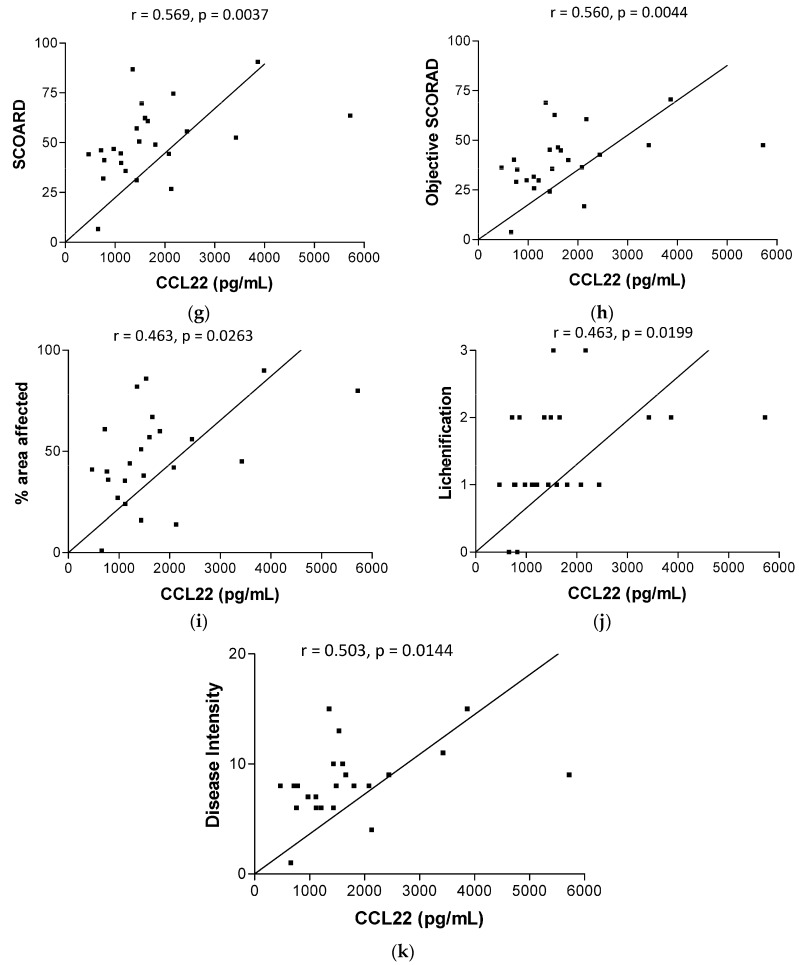
Correlations between plasma concentrations of CCL27 and CCL22 with SCORAD, objective SCORAD, % area affected, lichenification, disease intensity and pruritus in AD patients. Correlation and significant were determined by non-parametric Spearman’s correlation test.

Plasma concentrations of AD-related chemokine CCL27, CCL22 and CCL17 and antimicrobial peptides HNP and dermcidin were significantly higher in AD patients than in control subjects (median (IQR) of CCL27, CCL22, CCL17, HNP and dermcidin for AD patients *vs.* controls: 992.9 (754.1–1525) *vs.* 679 (519.6–751.5) pg/mL, *p* = 0.0005; 1435 (855–2105) *vs.* 598.2 (525.7–712.4) pg/mL, *p* < 0.0001; 83.6 (40.4–244.1) *vs.* 10.9 (8.7–25.8) pg/mL, *p* < 0.0001; 2245 (1863–3150) *vs.* 1783 (1412–2192) pg/mL, *p* = 0.0294 and 7.2 (5.9–8.1) *vs.* 5.4 (5.0–6.6) ng/mL, *p* = 0.003, respectively, all *p* < 0.05, Figure 1). As shown in Figure 2, plasma concentrations of CCL27 were found to be correlated significantly with SCORAD, objective SCORAD, % area affected, lichenification, disease intensity and pruritus (all *p* < 0.05), exhibiting strong correlation with % area affected and disease intensity (both *r* > 0.8). Plasma concentrations of CCL22 were also correlated significantly with SCORAD, objective SCORAD, % area affected, lichenification and disease intensity (all *p <* 0.05) with weaker Spearman's rank correlation coefficient comparing with that of CCL27.

CCL27/cutaneous T-cell-attracting chemokine (CTACK) is a T-cell attracting chemokine for the regulation of infiltration and accumulation of memory T lymphocytes to the skin, therefore, it plays a role in T cell-mediated skin inflammation in AD [25,26,27]. CCL27 produced by keratinocytes, gonads, thymus and placenta can bind to its receptor CCR10 on skin-homing T cells [27]. Previous studies showed the elevated CCL27 levels were significantly correlated with AD severity [25,26]. However, our correlation coefficient between CLL27 with SCORAD and objective SCORD was about 0.78 which is higher than the previously reported 0.68 [25]. CCL22/macrophage-derived chemokine (MDC) is secreted by dendritic cells and macrophages to elicit its effects on its target cells by interacting with cell surface chemokine receptors CCR4 [28]. CCL17/(thymus and activation regulated chemokine/TARC) is a basic protein which also acts on the chemokine receptor CCR4 on peripheral blood mononuclear cells (PBMC) and human T lymphocytes [29]. CCL17 could induce selective migration of lymphocytes, especially Th2 cells [29,30,31]. Therefore, CCR4-CCL22/CCL17 interaction is crucial in regulating the chemotaxis of Th2 lymphocytes into local inflammatory sites of allergic inflammation [30,32]. Elevated serum levels of CCL17 and CCL22 have previously been shown to be significantly correlated with the AD severity [33,34,35]. Together, our present results further confirmed the elevated crucial chemokines CCL17, CCL22 and CCL27 in AD, leading to the accumulation of Th2 cells and eosinophils/basophils and overexpression of Th2 cytokine IL-4, IL-10 and IL-13, and corresponding suppression of anti-microbial peptide expression at local skin lesions [36]. Therefore, the elevated production of chemokines eventually leads to enhanced *S. aureus*-mediated persistent skin inflammation which correlates with and exacerbates the AD disease severity. CCL27 and CCL22 can therefore serve as surrogate biomarkers for monitoring disease severity in AD.

Further analysis of the neutrophil-derived HNP and sweat glands-derived dermcidin showed that the plasma concentrations of HNP and dermcidin were significantly elevated in AD patients (all *p* < 0.05). Results therefore indicated that up-regulated circulating anti-bacterial innate immunity which is different from the down-regulated/defective anti-bacterial innate immunity at local skin lesions [23,24,37]. The impaired anti-*S. aureus-*mediated innate-immunity in skin may increase the access of bacteria into the circulation, thereby activating the systemic, especially neutrophil-mediated innate immunity, resulting in elevated circulating level of HNP and dermcidin. Deriving from the present clinical setting, results therefore indicated the dermcidin and HNP may play crucial roles for the regulation of circulating innate immunity in response to infection and injury in AD [17,19,38]. Since HDN and dermcidin can exert immunomodulating activity in innate immunity, their circulating elevation may participate in allergic inflammation in AD [17,19]. Other innate immunity-related molecules including LL37, neopterin and psoriasin however did not exhibit any significant difference between AD patients and controls subjects (all *p* > 0.05, data not shown).

### 2.3. Expression of TLR2 and NOD2 in CD203+ Granulocytes

**Figure 3:**
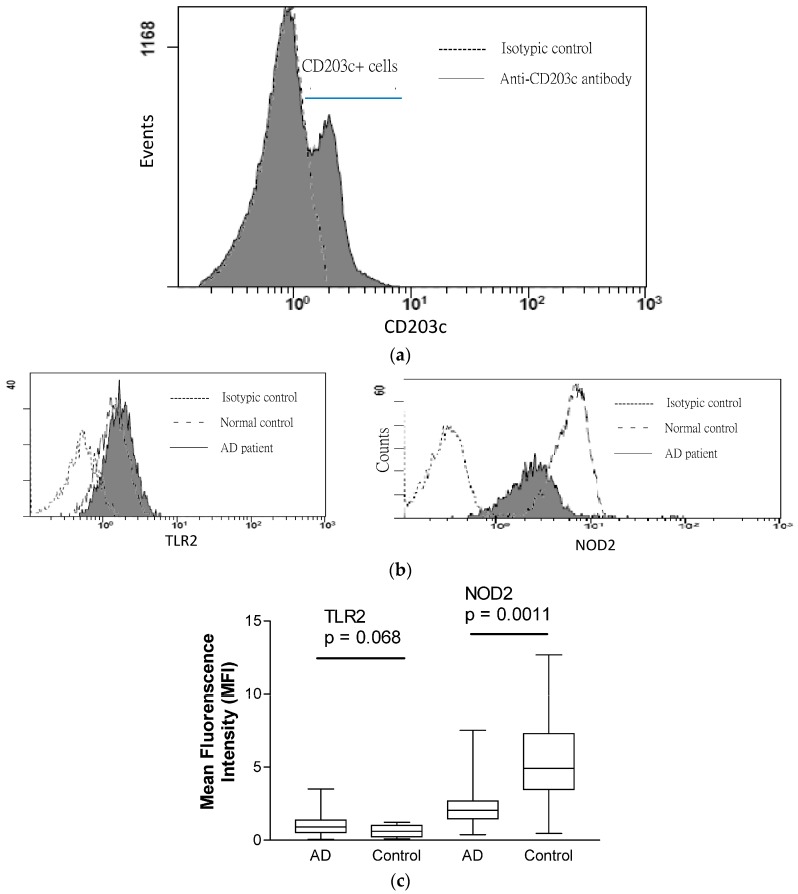
Expression of TLR2 and NOD2 in CD203-positive granulocytes. (**a**) Representative histogram is shown for the CD203+ cells gated from total granulocytes; (**b**) Representative histograms of the cell surface expression of TLR2 and intracellular expression of NOD2 of CD203c+ granulocytes are shown. (**c**) Box and whiskers plots showing the comparison of cell surface TLR2 and intracellular NOD2 expression (MFI) in CD203+granulocytes between AD patients and control subjects were determined by Mann-Whitney rank sum test. AD: patients with atopic dermatitis; Control: normal control subjects.

**Figure 4:**
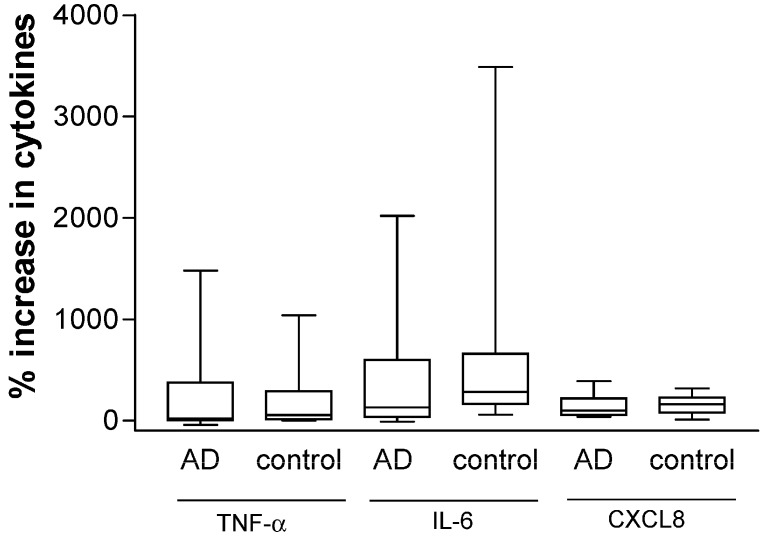
*Ex vivo* production of cytokines and chemokine from peripheral blood mononuclear cells (PBMC) of AD patients and control subjects upon NOD2 ligand MDP stimulation. PBMC (1 × 10^6^/mL) was incubated with or without NOD2 ligand MDP (1 μg/mL) for 24 h at 37 °C. After incubation, the cell free supernatant of *ex vivo* culture was used for the assay of inflammatory TNF-α, IL-6 and CXCL8 using human inflammatory cytokine cytometric bead array (CBA) kit. The % increase of the cytokine induction by MDP was calculated by (concentration of cytokines stimulated by ligand − basal concentration of cytokines without any stimulation)/basal concentration of cytokine without any stimulation × 100%. Results are presented with Box and whiskers plot. AD: patients with atopic dermatitis; Control: normal control subjects.

TLR2 and NOD2 can provoke cutaneous immune response against *S. aureus* by activating NF-κB-mediated production of AD-related chemokines and anti-microbial peptides [39], and basophils are the principal effector cells in allergic inflammation of AD [11]. In order to assess bacterial-infection related innate immunity in AD, we therefore investigated the expression of *S. aureus*-related PRR TLR2 and NOD2 in CD203+ basophils obtained from AD patients and controls. As shown in Figure 3a,b, the gated CD203+ granulocytes could denote the basophil population in total granulocytes and the expression of NOD2 and TLR2 of basophils. In Figure 3c, further analysis found that intracytosolic NOD2 but not cell surface TLR2 expression was significantly lower in AD patients than in control subjects (median (IQR) of NOD2 and TLR2 (MFI) for AD patients *vs.* controls: 2.04 (1.48–2.68) *vs.* 4.92 (3.47–7.30), *p* = 0.0011; 0.91 (0.53–1.39) *vs.* 0.62 (0.24–1.03), *p* = 0.068, respectively). Correspondingly, the % increase of the *ex vivo* induction of inflammatory cytokines TNF-α, IL-6 and CXCL8 upon NOD2 ligand MDP stimulation was found to be higher in PBMC culture from control subjects than that of AD patients (median (IQR) of % increase in TNF-α, IL-6 and CXCL8 for AD patients *vs.* controls: 20 (0–375) *vs.* 55 (15–290) %; 130 (35–600) *vs.* 285 (165–660) %; 100 (55–220) *vs.* 160 (80–225) %, respectively, Figure 4).

The above results therefore demonstrated the down-regulation of NOD2-mediated innate immunity of allergic inflammatory basophils in AD patients. Our recent findings have also demonstrated the down-regulation of NOD2 expression in eosinophils in patients with allergic asthma [40]. Together with the present results, bacterial PRR of granulocytes eosinophils and basophils seems to be impaired in allergy, linking the aberrant innate immunity with allergic inflammation in allergic diseases.

## 3. Materials and Methods

### 3.1. AD Patients, Non-Allergic Control Subjects and Blood Samples

Twenty-seven ethnic Chinese patients aged 9 to 18 years were recruited and managed in the pediatric dermatology outpatient clinics of a university teaching hospital in Hong Kong. The diagnosis of AD was made according to criteria proposed by Hanifin and Rajka [41]. The clinical severity of AD was assessed using the SCORAD index system (range 0–103) [42,43]. Skin biophysiologic parameters including skin hydration (SH) and transepidermal water loss (TEWL) were measured by standardized procedure following acclimatization to eliminate the seasonal effects on the measurements [43]. Fifteen corresponding non-allergic normal control subjects with similar age range were recruited. Control subjects did not have any history of allergic or inflammatory disease and did not require any drug treatment at the time of study. All recruited subjects were free from intercurrent illness including respiratory tract infection and allergic asthma, and they had not used any systemic corticosteroids for 4 weeks prior to enrollment in the study. The atopic status of these patients and control subjects was ascertained by positive serum-specific IgE assays to house dust mite *D. pteronyssinus*, cat dander, dog dander, mixed cockroaches, mixed molds and *Staphylococcal* enterotoxin A, B and C by fluorescence enzyme immunoassay (AutoCAP analyzer, Pharmacia Diagnostics AB, Uppsala, Sweden) [44,45,46] and basophil activation test [40]. *S. aureus* growth from wound swabs was obtained from AD patients to document current *S. aureus* colonization. Sixteen milliliters (mL) of EDTA-anticoagulated and 3 mL clotted venous peripheral blood were collected from each patient and control subject for analysis. Plasma and serum samples were preserved at −80 °C for subsequent assays. The above protocol was approved by the Clinical Research Ethics Committee of the Chinese University of Hong Kong-New Territories East Cluster Hospitals, and informed written consent was obtained from all subjects or their parents in accordance with the 1964 Declaration of Helsinki and its later amendments.

### 3.2. Flow Cytometric Analysis for TLR2 and NOD2 Expression in CD203+ Basophils from AD Patients and Controls

Peripheral blood granulocytes from AD patients and control subjects were purified using Percoll gradient centrifugation (GE Healthcare Life Sciences, Pittsburgh, PA, USA). The expression of TLR2 and NOD2 of CD203c+ granulocytes of AD patients and control subjects was determined by flow cytometry (Navios Flow Cytometer, Beckman Coulter Inc., Miami, FL, USA) as previously described [40,47]. Murine serum-blocked granulocytes were fixed and permeabilized using Fix/Perm solution (BD Biosciences Corp., San Jose, CA, USA) for the cell surface and intracellular staining of TLR2 and NOD2, respectively. Unconjugated mouse anti-TLR2 (Imgenex Corp., San Diego, CA, USA), anti-NOD2 antibody (Bio-legend Corp, San Diego, CA, USA), corresponding mouse IgG1, κ isotypic control antibody (BD Pharmingen Corp., San Diego, CA, USA), together with a fluorescein iso-thiocyanate (FITC)-conjugated goat anti-mouse IgG (H + L) secondary antibodies (Zymed Laboratories, Inc., South San Francisco, CA, USA), were used for staining. The expression of TLR2 and NOD2 of final granulocyte suspension with 10,000 events was assessed using flow cytometry with gating by CD203, a specific activation marker on basophils (Navios Flow Cytometer). Results were expressed as mean fluorescence intensity (MFI).

### 3.3. Quantitative Analysis of Plasma Concentrations of Chemokines, Dermcidin and HNP

Plasma concentrations of chemokines were measured by Bio-plex human cytokine multiplex assay with the Bio-plex 200 System (Bio-Rad Laboratories, Inc., Hercules, CA, USA), while plasma concentrations of dermcidin and HNP were measured by enzyme-linked immunosorbent assay (ELISA) reagents from MyBiosource, San Diego, CA, USA, and Hycult Biotech Inc., Plymouth Meeting, PA, USA, respectively.

### 3.4. Assay of Serum IgE

Serum specific IgE against house dust mite (Der p1), cat and dog dander, mixed cockroaches, mixed molds, Staphylococcal enterotoxin A, B and C were measured to ascertain the atopic status of all participants using fluorescence enzyme immunoassay (ImmunoCAP immunoassay analyzer, Phadia, Uppsala, Sweden) [46,48].

### 3.5. Basophil Activation Test (BAT)

To further assess the IgE-mediated allergic inflammation, we evaluated the functional basophil-bound-specific IgE using specific allergen-mediated BAT (Allergenicity Assay Kit, Beckman Coulter). This is a cellular functional analysis based on flow cytometric analysis of the IgE-mediated up-regulation of the cell surface expression of activation marker CD203c associated with cellular degranulation on basophils upon the *ex vivo* allergen challenge such as house dust mite, cockroaches, and cat dander [40,49]. Using an accurate basophil gating tool (CRTH2+ CD203c+ CD3−), BAT was performed on whole blood specimen by flow cytometer (Navios Flow Cytometer, Beckman Coulter).

### 3.6. Ex Vivo Induction of Inflammatory Cytokines from PBMC

After a maximum storage period of 1 h of freshly collected EDTA blood at room temperature, PBMC were purified using Ficoll-Paque gradient centrifugation (GE healthcare Life Sciences, NJ, USA). PBMC (1 × 10^6^/mL) was then incubated with or without NOD2 ligand muramyl dipeptide (MDP, 1 μg/mL) (Invivogen Corp, San Diego, CA, USA) for 24 h at 37 °C in a 5% CO_2_ atmosphere. After incubation, the cell free supernatant of *ex vivo* culture was harvested and stored at −70 °C for subsequent assay of inflammatory cytokines using human inflammatory cytokine cytometric bead array (CBA) kit with the FACSCalibur flow cytometer (Becton Dickinson, San Jose, CA, USA) [50]. The % increase of the cytokine induction by PGN and MDP was calculated by (concentration of cytokines stimulated by ligand − basal concentration of cytokines without any stimulation)/basal concentration of cytokine without any stimulation × 100%.

### 3.7. Statistical Analysis

The non-parametric Mann-Whitney rank sum test was used to compare the differences between different groups, and the Spearman’s rank correlation test was used to assess the correlations among different measurable parameters. All analyses were performed using Statistical Package for the Social Sciences (SPSS) statistical software for Windows, Version 20 (SPSS Inc., Chicago, IL, USA). A probability *p* < 0.05 was considered as statistically significant.

## 4. Conclusions

Elevated circulating Th2 chemokines are related to the infiltration of Th2 cells and the accumulation of eosinophils/basophils with the inhibition of the expression of anti-microbial peptides at skin lesions. These lead to worsening symptoms and increased disease severity scores in AD. Therefore, the impaired anti-bacterial innate immunity has been shown to account for the enhanced skin bacterial infections in AD [23,24,37]. In the present study, the defective PRR have been identified for allergic inflammatory basophils. Together with lower cytokine response upon NOD2 ligand stimulation and lower concentration of anti-microbial peptides HNP and dermcidin at local skin lesions, the local innate immune mechanisms against *S. aureus* could be down-regulated in AD. This may subsequently lead to enhanced *S. aureus* proliferation at skin lesions, and bacteria or its pattern associated molecular pattern molecules may penetrate into the blood circulation to stimulate the neutrophils and other immune effector cells-mediated innate immunity in circulation to result in increased plasma levels of HNP and dermcidin. The present study may provide further evidence for the elucidation of these defects of innate immunity and allow the development of novel therapeutic intervention to repair these deficiencies and reduce the allergic inflammation in AD patients.

## Figures and Tables

**Table 1:** Demographic, clinical and laboratory data of the atopic dermatitis (AD) patients.

Parameters	AD Patients	Control Subjects
	(*n* = 27)	(*n* = 15)
Sex (male/female)	15/12	5/10
Age at evaluation, year (mean ± SD, range)	14.3 ± 2.4 (9–18)	21.2 ± 6.1 (10–29)
SCORAD index	48.0 (40.5–61.7)	N.A.
Objective SCORAD index	38.2 (29.9–47.0)	N.A.
Intensity (*Erythema, Edema/Papulation, Oozing/Crusting, Excoriation, Lichenification Dryness*)	8.0 (6.5–10.0)	N.A.
Pruritus	7.0 (4.5–8.0)	N.A.
Sleep loss	5.5 (0.0–7.5)	N.A.
SH	24.6 (15.4–34.7)	N.A.
TWEL	8.5 (7.8–10.8)	N.A.
Area affected (%)	44 (35.8–64.0)	N.A.
Lichenification	1.0 (1.0–2.0)	N.A.
CDLQI	10.0 (4.5–15.5)	N.A.
Presence of at least one positive allergen-specific IgE, *n* (%)	27 (100)	0 (0)
*D. pteronyssinus*, *n* (%)	27 (100)	0 (0)
Cockroaches, *n* (%)	16 (59)	0 (0)
Cat dander, *n* (%)	13 (48)	0 (0)
Dog dander, *n* (%)	21 (78)	0 (0)
Mixed molds, *n* (%)	18 (67)	0 (0)
*Staphylococcal* enterotoxin A, *n* (%)	16 (59)	0 (0)
*Staphylococcal* enterotoxin B, *n* (%)	14 (52)	0 (0)
*Staphylococcal* enterotoxin C, *n* (%)	12 (44)	0 (0)
Positive basophil activation test		
House dust mite, *D. pteronyssinus*, *n* (%)	26 (96)	0 (0)
Cockroaches, Bla g 2 protein, *n* (%)	21 (78)	0 (0)
Cat dander, Fel d 1 protein, *n* (%)	19 (70)	0 (0)
Positive *S. aureus* growth (wound swabs), *n* (%)	18 (70)	N.A.
Treatment		
Topical corticosteroid	24 (89)	N.A.
Emollient	27 (100)	N.A.

Values were given as median (IQR) or mean ± SD. SCORAD: SCORing atopic dermatitis, N.A.: not applicable, SH: skin hydration, TWEL: transepidermal water loss, CDLQI: Children’s Dermatology Life Quality Index.
